# Multidisciplinary team approach to the prenatal management of orofacial clefts: a single center cohort study in Taiwan

**DOI:** 10.1038/s41598-020-70906-1

**Published:** 2020-08-18

**Authors:** Hsuan Ko, Tung-Yao Chang, Eric C. Lussier, Ksenia Olisova, Chan-Yu Sung, Philip Kuo-Ting Chen, Wen-Chu Li, Tze-Yi Yang, Ru-Xuan Wang

**Affiliations:** 1 Taiji Clinic, Taipei, Taiwan; 2 Taipei Medical University Hospital, Taipei, Taiwan; 3 Puli Christian Hospital, Puli Township, Taiwan

**Keywords:** Ultrasonography, Health services, Intrauterine growth

## Abstract

Advances in ultrasound fetal diagnostics and treatment have created a dilemma for doctors and parents: choosing whether to continue with a pregnancy as well as choosing between various treatment options. A multidisciplinary approach has been widely accepted in the management of other prenatally diagnosed anomalies and has shown superior results compared to routine care. We present a retrospective cohort of patients prenatally diagnosed with orofacial clefts who were offered consultation by an expert multidisciplinary team, including: a fetal medicine specialist, an obstetrician, a plastic surgeon, and a case managing nurse. We analyzed factors influencing parents' decision to utilize a consultation service, as well as their decision about pregnancy continuation. Our results suggest that the presence of other anomalies and maternal age heavily influenced the decision about the uptake of consultations. If consulted by the team, parents tended to continue with the pregnancy, even when accounting for fetal gender and maternal age. On the other hand, having a consultation had varying effects depending on the cleft type. The findings suggest that multidisciplinary consultations may be an efficient approach in managing pregnancies complicated by orofacial cleft anomalies; which may help in preventing unnecessary pregnancy terminations and developing a sufficient postnatal care plan.

## Introduction

An orofacial cleft (OFC) is a common congenital structural anomaly that occurs in about 1/500 to 1/2,500 of live births^1^. The incidence of cleft lip and palate is the highest among Asian populations^2^. Children with OFCs are confronted with not only having an abnormal facial appearance, but also with the sequelae of OFCs, such as: hearing loss, vocalization disability, difficulty in chewing, etc^3^.

In a previous qualitative study of expectant mothers who had received a prenatal OFC diagnosis in Taiwan, they reported various psychosocial impacts, including: loss of self-worth, decision-making conflicts, anxiety caused by insufficient information, and economic burden^4^. In Taiwan, when a fetal anomaly is diagnosed, women are legally allowed to choose termination before 24 weeks^5^. Sufficient access to appropriate and comprehensive information before 24 weeks can decrease the rate of unnecessary terminations.

Prenatal counseling allows parents to be provided with sufficient knowledge and for parents and physicians to establish a long-term treatment plan^6^.Counseling sessions have been found to be effective in easing parents’ anxiety that might follow a prenatal diagnosis^7^. Recent advances in diagnostic technology and techniques have led to a higher sensitivity of fetal ultrasonography for detecting facial clefts with other structural anomalies in Taiwan^8^. Due to the advanced detection rate, an opportunity has emerged to create a multidisciplinary team that can assist parents to identify their optimal postnatal treatment options^9^. Furthermore, although a multidisciplinary approach has been shown to be efficient in managing abnormalities, the vast majority of previous studies have focused on postnatal abnormalities. Therefore, research about multidisciplinary approaches to prenatally diagnosed OFC management is limited. In this study, we described the characteristics and the rate of pregnancy continuation in a retrospective cohort of prenatally diagnosed OFC cases that had been offered multidisciplinary team consultation services.

## Results

### Demographic characteristics

A total of 39 OFC cases were included in this retrospective cohort upon retrospectively reviewing clinical visit record data. Descriptive statistics are reported in Table 1. The majority of cases were male (n = 25, 64.1%), while two cases had an unknown sex due to being diagnosed with multiple anomalies before 12 weeks and subsequently being terminated before 17 weeks. The incidence of unilateral OFC (n = 29, 74.4%) was higher than bilateral (n = 8, 20.5%). Furthermore, most cases were isolated clefts without other abnormalities (n = 34, 87.2%).

**Table 1:** Characteristics of the study sample (n = 39).

Variables	Groups	n	%
**Fetal sex**			
	Female	12	30.8
	Male	25	64.1
	Unknown	2	5.1
**Orofacial cleft type**			
	Unilateral	29	74.4
	Bilateral	8	20.5
	Type 4	1	2.6
	Type 5	1	2.6
**Other abnormalities**			
	No	34	87.2
	Yes	5	12.8
**Orofacial cleft consultation**			
	No	13	33.3
	Yes	26	66.7
**Decision to continue with pregnancy**			
	Discontinued	15	38.5
	Continued	24	61.5
**Maternal age, years**			
	> 30	22	56.4
	≤ 30	17	43.6

Data represented by count (n) and percentage (%).

### Consultation uptake

In Table 2, case characteristics were compared by acceptance of OFC consultations. Cases that had another abnormality besides having an OFC tended not to participate in the OFC consultation (*p* value = 0.035). Maternal age played a significant role (*p* value = 0.005) in having a multidisciplinary team consultation or not. Older expectant mothers had a higher acceptance rate compared to younger mothers. All other characteristics failed to reach statistical significance.

**Table 2:** Study sample characteristics by consultation uptake (n = 39).

Variables	Groups	Consultation received				Fisher’s exact test
		No		**Yes**		
		n	%	n	%	*p*
**Fetal Sex**						1.000
	Female	3	23.1	9	34.6	
	Male	8	61.5	17	65.4	
	Unknown	2	15.4	0	0.0	
**Oral cleft severity**						0.862
	Unilateral	11	84.6	18	69.2	
	Bilateral	2	15.4	6	23.1	
	Isolated cleft palate	0	0.0	1	3.8	
	Submucous cleft palate	0	0.0	1	3.8	
**Other abnormalities**						0.035*
	No	9	69.2	25	96.2	
	Yes	4	30.8	1	3.8	
**Maternal Age, years**						
	≤ 30	10	76.9	7	26.9	0.005*
	> 30	3	23.1	19	73.1	

Data represented by count (n) and within column percentage (%). Univariate analysis by Fisher’s exact test, significance levels *p < 0.05; **p < 0.01; ***p < 0.001.

### Pregnancy outcome

**Figure 1:**
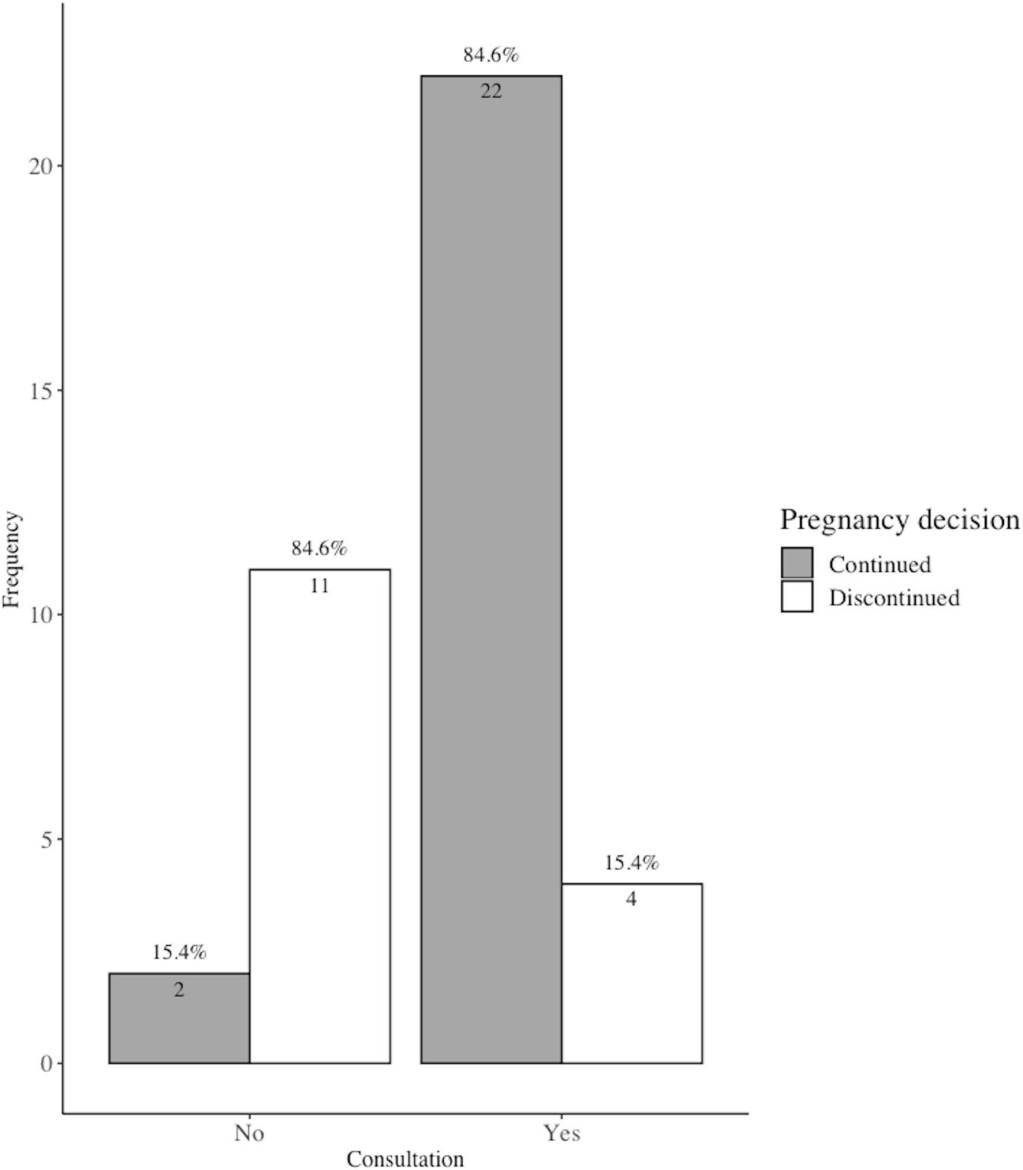
Pregnancy decision outcome by consultation uptake following an orofacial cleft diagnosis. Univariate analysis by Fisher’s exact test, statistically significant at *p* value < 0.05.

Parent’s decision of whether to have a prenatal OFC consultation and to continue with the pregnancy were compared (Fig. 1). Our findings showed that parents who had an OFC consultation had a significantly higher pregnancy continuation rate than parents who had not had a consultation (84.6% vs. 15.4%, *p* < 0.001).

### Subgroup analysis

Subgroup analysis of pregnancy continuation rates by antenatal characteristics and utilization of the consultation are shown in Table 3. Among mothers who utilized the consultation service, there was a tendency towards pregnancy continuation for both male and female fetuses. A similar trend of pregnancy continuation was observed for women below and above 30 years of age, as there was a trend to continue the pregnancy for both age groups after having a consultation. Cases with unilateral OFC saw higher rates of continuation if they had a consultation, in contrast those with a bilateral type did not differ significantly in their pregnancy continuation rate. Isolated cases who received a consultation had higher rates of continuation compared to isolated cases who chose not to receive a consultation, however, there was no considerable difference in continuation for the group with multiple anomalies.

**Table 3:** Subgroup analysis of pregnancy decision by antenatal characteristics and consultation uptake (n = 39).

			Continuation		Fisher’s exact test
Variables	Groups	Consultation	Continued/total n	Continuation rate (%)	*p*
**Gender**					
	Female	No	0/3	0.0	0.045*
		Yes	7/9	77.8	
	Male	No	2/8	25.0	0.004**
		Yes	15/17	88.2	
	Unknown	No	0/2	0.0	n/a
		Yes	0/0	0.0	
**Orofacial cleft type**					
	Unilateral	No	2/11	18.2	< 0.001***
		Yes	16/18	88.9	
	Bilateral	No	0/2	0.0	0.429
		Yes	4/6	66.7	
**Other anomalies**					
	No Other Abnormality	No	2/9	22.2	0.002**
		Yes	21/25	84.0	
	Other Abnormality	No	0/4	0.0	0.2
		Yes	1/1	100.0	
**Maternal age, years**					
	> 30	No	0/3	0.0	
		Yes	16/19	84.2	0.01*
	≤ 30	No	2/10	20.0	
		Yes	6/7	85.7	0.02*

Univariate analysis by Fisher’s exact test, significance levels *p < 0.05; **p < 0.01; ***p < 0.001.

## Discussion

Multidisciplinary consultation provides mental support and education to parents regarding steps in OFC care, including fetal anomaly type evaluation, prognosis, and the success rate of postnatal surgery, consequently relieving parental anxiety and guilt^10^. Previously, it has been shown that a multidisciplinary approach can result in improvements in prenatal management, diagnosis, treatment and postnatal care, which can allow for better coordination between different health care teams and has resulted in the establishment of alternate treatment guidelines^11^. For these reasons, we believe that the utilization of prenatal multidisciplinary care can improve parents' perception of prenatal OFC diagnosis and decrease the rates of unnecessary pregnancy terminations. As we have shown in our study, having multiple abnormalities and maternal age above 30 years were two main factors associated with parental consultation uptake. Furthermore, having access to a multidisciplinary consultation was significantly associated with higher pregnancy continuation rate compared to the parents who did not receive consultation and further having consultation led to significant improvements in continuation rates regardless of fetal gender or maternal age, and was especially beneficial for unilateral cleft type.

We found that cases that had an additional anomaly to OFC were less likely to agree to having a consultation. A similar trend was found in a previous study among mothers who had received a prenatal upper extremity anomaly diagnosis, having additional anomalies, or a more severe cases, were less likely to agree to have a consultation^12^. One possible explanation for having fewer consultations, was that parents had likely considered the difficulties in the treatment of children with multiple anomalies and their subsequent life course. Often, a decision about pregnancy continuation is made before psychologic feto-maternal bonding is formed^13^. Thus, making them more likely to choose not to elect to undergo a consultation. We demonstrated that maternal age correlated to a higher rate of the uptake of a multidisciplinary consultation in our sample. Mothers aged over 30 years old had a higher rate of OFC consultations compared to their younger counterparts. As described previously, mothers of advanced age are more likely to have a higher education, socio-economic status and level of knowledge regarding pregnancy risks^14,15^, which may have influenced their decision-making. Another possibility is that women of advanced ages were more likely to choose a consultation due to them having a higher chance of perceiving that this might be their last pregnancy^16^.

Overall, there was a significant association between consultation uptake and increased continuation rate. Patients having a consultation had seen a higher rate of pregnancy continuation. As was previously shown, employing a multidisciplinary approach allows for: improvement in accuracy of ultrasound diagnosis, access to the comprehensive information, and support from the multidisciplinary team, which has been previously shown to help relieve mental burden, alleviate anxiety and improve likelihood of informed decision making by parents^10,17,18^. Although, our sample size is small, we believe that multidisciplinary consultation played an important role in increasing the continuation rates in pregnancies diagnosed with OFC. Our findings are in line with the recent expansion of using a multidisciplinary approach to care^8,9,19^, thus we support employing a multidisciplinary approach to OFC care.

A higher rate of pregnancy continuation was observed for cases that had a consultation compared to patients that had refused a consultation. Consultations appeared to improve continuation for unilateral cleft type, for cases without other anomalies, with improvements in continuation appearing regardless of fetal gender and maternal age. It is expected, that isolated cases would benefit more from the consultation than cases with multiple anomalies^3^ and that their pregnancy continuation rate would be high, although in our sample we saw a high rate of pregnancy termination among isolated cases, 11/34 (32.4%) pregnancies with isolated OFC were terminated. Moreover, there is a high rate of pregnancy termination and sex-selective abortion with a tendency to keep male fetuses in Taiwan^21^ due to cultural views and accessibility of the healthcare. Furthermore, despite older mothers being more likely to use consultation, women aged above or below 30 years appeared to be both equally benefited by having a consultation. Despite these cultural and age characteristics, we saw a significant correlation between having a consultation and pregnancy continuation for both female and male fetuses and both maternal age groups, further strengthening support for implementing a multidisciplinary approach to OFC care.

It is necessary to consider the study limitations when generalizing our findings. First, our study was a retrospective cohort and the outcomes were known, which can introduce some bias into our results. Second, due to the small sample size, our study provides a snapshot of the effect following an OFC consultation. Further research can build on our findings to further explore how the multidisciplinary approach influences parental decision making in prenatal cleft diagnosis cases. Third, our health center is a private clinic, parents were mainly from Taipei City, thus our results may differ from the general population which likely has different socio-economic characteristics. A more representative sample is needed to fully explore the effects of a multi-disciplinary team. Fourth, our clinic is a specialized fetal care center, and the majority of patients being referred have a suspected anomaly. Our sample, therefore, does not reflect the general population, and our results should be generalized to the larger population with caution. Nonetheless, our study provides evidence that the multidisciplinary approach to care at a specialized clinic can be of benefit to prenatally diagnosed OFC cases, and their subsequent consultation and pregnancy decision.

The goal of prenatal care is to detect abnormal cases, and for cases with positive findings to be provided with the necessary information regarding the anomaly, and to be given a better understanding of treatment options and life course of their unborn child. A multidisciplinary team can fully inform parents about all aspects of prenatal and postnatal care for babies with an OFC, which can assist parents in making an informed decision regarding pregnancy continuation. A well-organized team should consist of an experienced ultrasonographer, obstetrician, fetal medicine specialist, a plastic surgeon and a case manager; which in some contexts may be difficult to assemble. Therefore, referral to specialized health care facilities with capabilities of providing the necessary personnel and support for the family may be merited. In the future, a multidisciplinary approach should be widely utilized to ensure the best quality of care for families facing the hardships of dealing with a prenatal OFC diagnosis.

## Methods

### Sample

A cohort of 39 fetuses was retrospectively reviewed at a private primary care screening center that specializes in fetal ultrasound medicine. Patients were selected by comprehensively searching records of cases that had an OFC diagnosis and had been offered OFC consultation from our clinic between December 2017 and June 2019. Expectant mothers who attended an advanced mid-pregnancy fetal anatomical screening by self-referral or had been referred for further examination by a local obstetrician due to suspected OFC, were included in the study. All confirmed OFC cases had been suggested to undergo karyotyping at the time of diagnosis. With exception of fetuses with multiple anomalies, the majority of the expectant mothers did the testing, and only one case had a chromosomal microdeletion, but the fetus had no other abnormalities except for an OFC. Other abnormalities included severe systemic anomalies which might be lethal, such as: cardiac anomaly, renal anomaly, and congenital diaphragmatic hernia. Moreover, birth outcomes were obtained by reviewing hospital records and/or by phone interview. Due to the retrospective observational nature of the study, the requirement for informed consent was waived by the ethics committee which approved the study. Ethics review was conducted by the Institutional Review Board at Taipei Medical University (IRB #: N201910062). All methods were performed in accordance with the relevant guidelines and regulations.

### Ultrasound examination

**Figure 2:**
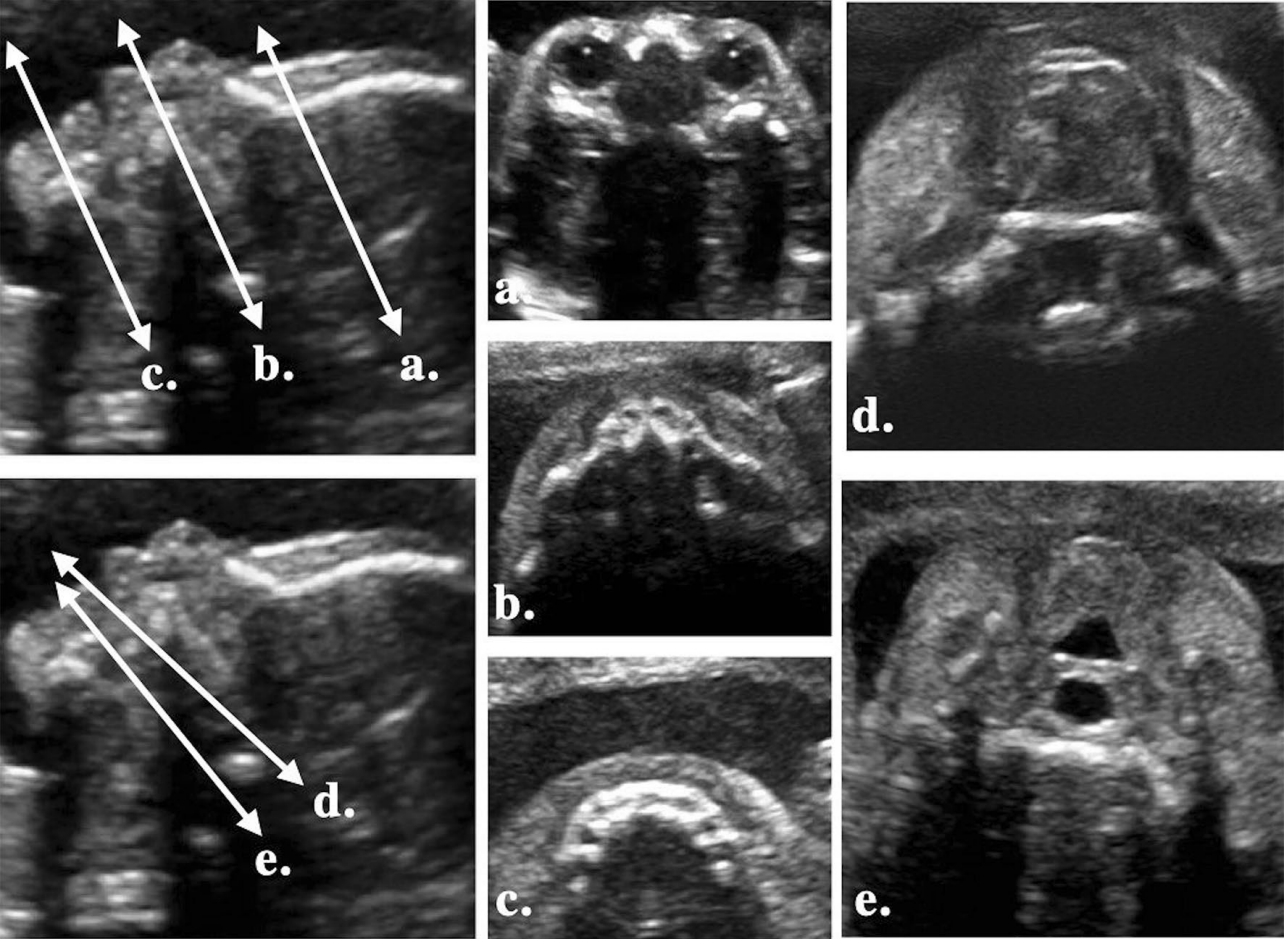
Ultrasound stepped views. To confirm the OFC diagnosis, 5 planes were obtained. (**a**) Eye sockets (**b**) upper alveolus (**c**) lower alveolus (**d**) posterior edge of hard palate (**f**) inferior part of soft palate.

Cases confirmed to have an OFC by fetal medicine specialist were referred to the clinic to be re-examined by our multidisciplinary team, which included: an experienced radiological technician, an obstetrician with expertise in 3D ultrasound, a fetal medicine specialist, a plastic surgeon, and a case managing nurse. The technician checked fetal face in stepped views, as displayed in Fig. 2. Conventional planes were used to assess the symmetry of the eye sockets, the upper alveolus, and the lower alveolus included in planes a, b and c. To further visualize the posterior edge of the hard palate and inferior part of the soft palate, planes d and e were obtained by tilting the transducer towards the fetal chest.

OFC typing for case management was based on the classification system proposed by Maarse et. al using “u” for unilateral, and “b” for bilateral type^20^. Overall, there are eight categories of OFC types. Degree of severity ranges from Type 1 to Type 3. Type 1 only affects the lips, type 2 affects the lips and alveolus, and Type 3, is the most severe type, affecting the lips, alveolus and palate; Types 4 and 5 are not likely diagnosed prenatally due to normal outer oral anatomy. Although, for the statistical analysis we simplified the classification only for unilateral and bilateral types.

### Orofacial consultation

**Figure 3:**
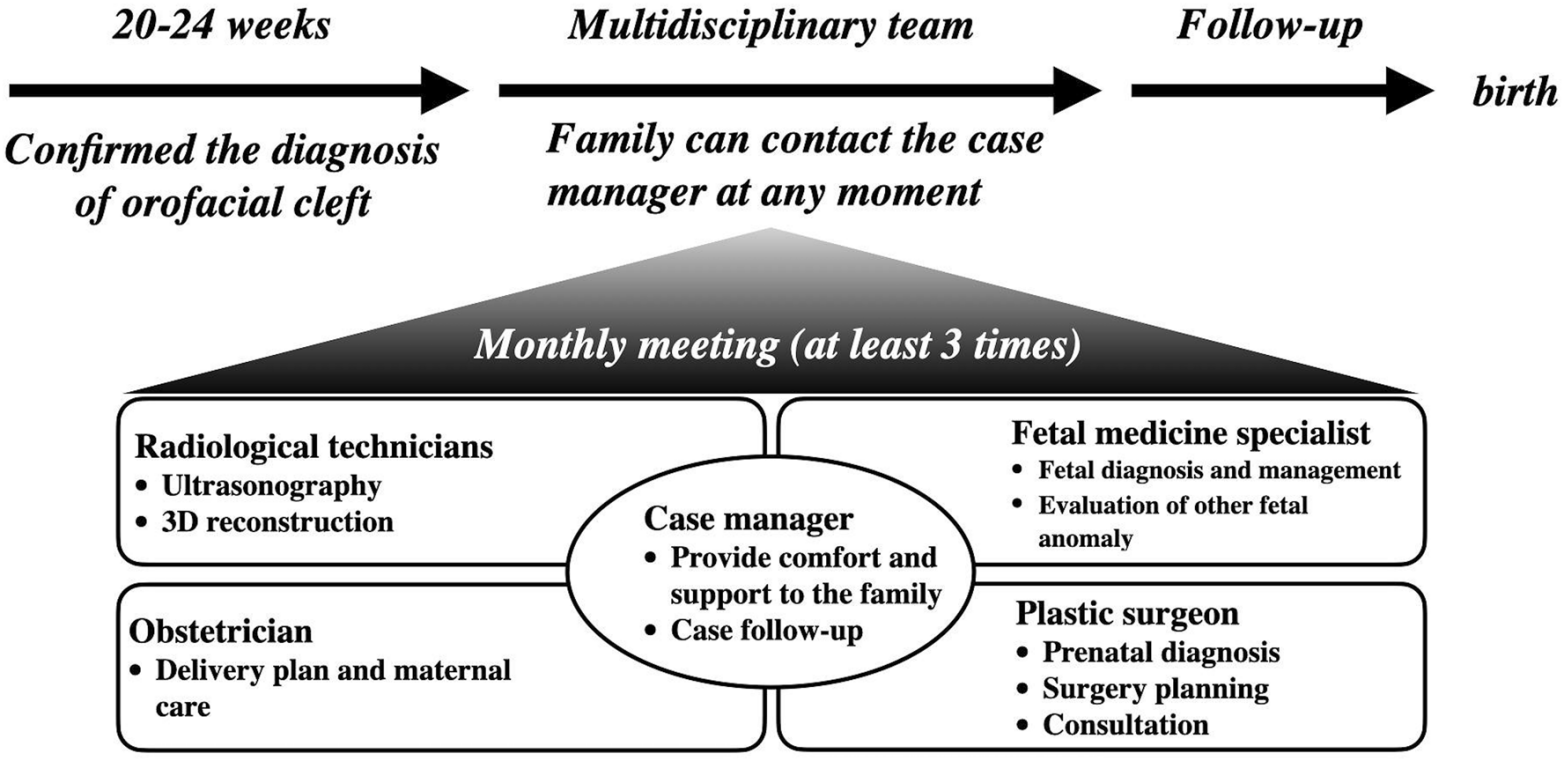
Flow diagram of multidisciplinary management. Flow of the multidisciplinary prenatal care.

The roles of multidisciplinary team members are shown in Fig. 3. If parents were willing to accept consultation, they were given the essential information regarding health, postpartum surgery options, and a child’s life course following the surgery. The case managing nurse was responsible for contacting parents, timely prenatal consultation, and providing comfort and support. If the parents struggled with the decision regarding pregnancy continuation, the case manager was active in following-up. To ensure no additional psychological harm was caused to parents, consultations were worded with tact, and instead of pushing for pregnancy continuation, the main aim was to ensure they had access to sufficient information to make an informed decision, and to support parents in their choice.

### Analysis

Descriptive statistics were used to characterize the OFC cases. Frequency was described by count (n) and percentage (%). A comparison between case characteristics and the decision to use consultation services, as well as a comparison between consultation services and the decision of whether to continue with pregnancy was performed. Due to some cells having a small count of less than n = 5, a Fisher’s Exact Test (*X*^2^) test of independence was performed. An a priori cut-off of significance was set at *p* < 0.05. All analyses were performed using R (RStudio Team (2019). RStudio: Integrated Development for R. RStudio, Inc., Boston, MA. URL https://www.rstudio.com/).

## Data Availability

The dataset analyzed in this study are not publicly available due to patient confidentiality concerns, but could be made available upon reasonable request to the corresponding author.
